# Modeling of respiratory network: to sigh or not to sigh

**DOI:** 10.1186/1471-2202-16-S1-P48

**Published:** 2015-12-18

**Authors:** Tatiana Dashevskiy, Jan-Marino Ramirez

**Affiliations:** 1 Center for Integrative Brain Research, Seattle Children's Research Institute, Seattle, WA, USA; 2 Department of Neurological Surgery, University of Washington, Seattle, WA, USA

## 

The Pre-Bötzinger Complex (PreBötC), a medullary brainstem region, is essential for generating breathing. Isolated in a transverse slice preparation, the preBötC continues to generate under normal conditions "fictive eupneic activity" and at a lower frequency also "fictive sighs". The mechanisms generating the periodicity of fictive sighs as well as its intrinsic nature are largely unknown. Based on the experimental observations such as 1) sigh has the biphasic shape characterized as eupneic breath interrupted by augmentation with a second peak of activity, 2) the biphasic shape persists after blockage of inhibitory synapses, 3) the network is heterogeneous, we model PreBötC network that reproduces both types of activities. The model contains three types of neurons: intrinsically tonic spiking, intrinsic bursting neurons and intrinsic quiescent. Neurons within the network are sparsely connected through randomly distributed excitatory synapses. In this model we propose that the mechanism of sigh generation is based on slow oscillations generated by glia. Moreover, the model predicts that depending on the modularity state of the system, the network exhibits sighs, does not exhibit sighs and shows only eupneic activity, or shows bi-stable activity meaning that sighs can be triggered between "on" and "off" states by short perturbation. The bistability depends on slow calcium oscillations. The network model predicts that the presence of IP3-like channels is necessary to produce "sigh" behavior and to mimic transitions between activity states caused by neuromodulators. We analyze the model dynamics and describe dose-response curve to neuromodulator such as norepinephrine. Theoretical data are similar to experimental data from *in vitro *brain slice preparations and *in vivo *anesthetized mice.

